# Successful treatment of corneal wasp sting-induced panuveitis with vitrectomy

**DOI:** 10.1186/1869-5760-3-18

**Published:** 2013-01-21

**Authors:** Yusuke Nakatani, Akira Nishimura, Kazuhisa Sugiyama

**Affiliations:** 1 Department of Ophthalmology and Visual Science, Graduate School of Medical Science, Kanazawa University, 13-1 Takara-machi, 920-8641, Kanazawa, Japan; 2 Department of Ophthalmology, Himi Municipal Hospital, 935-8531, Himi, Japan

**Keywords:** Corneal wasp sting, Endothelial cell analysis, Panuveitis, Vitrectomy

## Abstract

**Background:**

This study aims to present the management and clinical findings of a case of corneal wasp sting and to report the outcome of corneal change and panuveitis after vitrectomy.

**Findings:**

Clinical findings, anterior segment photographs, corneal endothelial changes, and medical treatment of corneal wasp sting-induced panuveitis are presented. A 95-year-man was stung by a wasp on his left cornea. A severe conjunctival hyperemia, marked corneal edema, corneal epithelial defect, and uveitis developed. As soon as the patient visited our clinic, topical corticosteroid and antibiotics were given, but corneal endothelial damage and uveitis did not improve. Anterior chamber irrigation was performed with oxiglutatione solution to rinse out the wasp venom. Corneal edema and anterior uveitis improved but the endothelial cell density gradually decreased and the vitreous opacity deteriorated. Therefore, a 23-gauge vitrectomy was performed. Subsequently, the corneal edema and panuveitis improved.

**Conclusions:**

Vitrectomy may be an effective treatment for corneal endothelial damage and endophthalmitis induced by a corneal wasp sting.

## Findings

### Introduction

Wasp sting of the cornea is a relatively rare injury. Various cases describing corneal bee or wasp stings have been reported. Wasp venom contains up to 13 different antigens. Serotonin causes multiple effects through 5-hydroxytryptamine (5-HT) receptors, including an intense localized vascular spasm. In addition, wasp venom contains phospholipase A, phospholipase B, and mastoparan peptide, which can cause direct mast cell degranulation with the release of histamine. Sudden release of histamine in the venom causes vasodilatation, an increase in capillary permeability, and a type 1 hypersensitivity immunological response mediated by immunoglobulin E [1-7]. Clinical course and prognosis ranged from mild to severe damage depending on the type of bee or wasp, depth of injury, and toxic or immunological response to the venom injected by the stinger. Complications reported in the literature were corneal edema, corneal infiltration, anterior uveitis, hyphema, cataract, lens subluxation, and optic neuritis. We report a case of corneal wasp sting which induced a corneal epithelial defect, endothelial damage, anterior uveitis, and *panuveitis*. To our knowledge, there is a paucity of the reports on the improvement of panuveitis induced by wasp sting after vitrectomy. This research was carried out with the ethical approval of Himi Municipal Hospital.

### Case report

A 95-year-old man consulted our in hospital with complaint of severe pain resulting from a wasp sting of the left eye that occurred during a logging operation. The patient was examined 1 h after the trauma. Visual acuity corrected by refraction was at the level of hand motion in the left eye and 20/20 in the right eye. There was no significant pathology in the right eye. He had undergone phacoemulsification and intraocular lens implantation of both his eyes 1 year previously. Visual acuity corrected by refraction of the left eye was 20/20, and the endothelial cell density was 1,753 cell/mm^2^ after cataract surgery.

**Figure 1:**
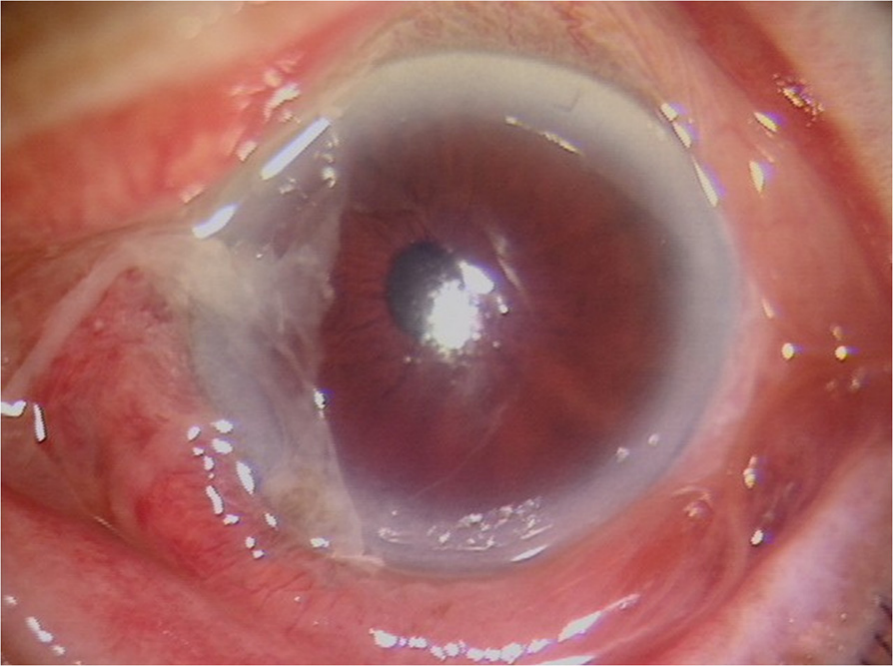
**Slit lamp photography at initial presentation.** Slit lamp photography at initial presentation showed conjunctival hyperemia, slight corneal edema, and a corneal epithelial defect.

Slit lamp examination of the left eye showed severe chemosis and conjunctival hyperemia (Figure 1). The cornea was slightly edematous and showed a partial epithelial defect after dyeing with fluorescein. No leakage was observed and the wasp stinger was not found. The anterior chamber showed normal depth with +2 cells and was flared. Hypopyon and posterior synechia were not found. Fundus examination could not be performed due to corneal edema. No relative afferent pupillary defect was observed. He had an unremarkable medical history and no underlying illness. We obtained consent of the subject.

**Figure 2:**
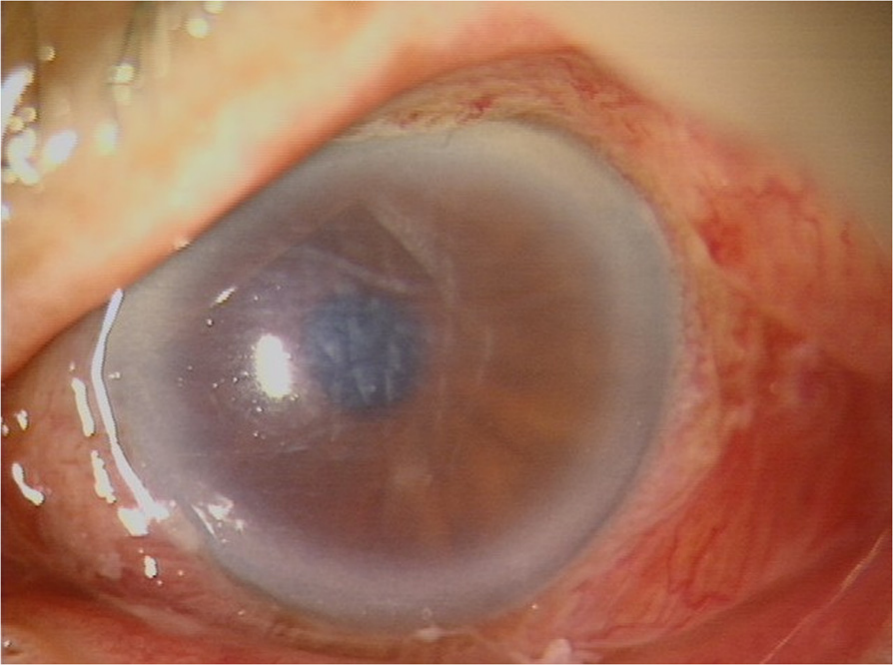
Slit lamp photography showed that corneal edema increased with Descemet’s membrane folds 3 days after injury.

**Figure 3:**
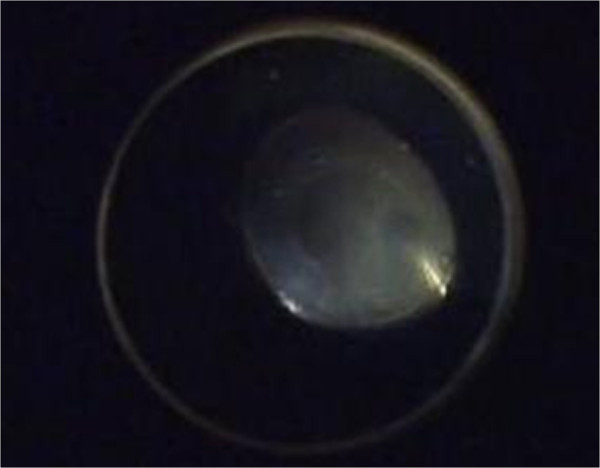
**Intraoperative view of 23-gauge sutureless vitrectomy.** Note the whitish vitreous opacity before the vitrectomy.

The patient was initially treated with topical antibiotics (ofloxacin, 0.3% at 6 times per day), topical steroid (dexamethasone, 2 mg/day at 6 times per day), and tropicamide (3 times per day). Subconjunctival steroid injections (dexamethasone, 2 mg/day for 2 days) were given until resolution of the epithelial defect. On the third day of treatment, the corneal epithelial defect decreased, but the corneal edema deteriorated with Descemet’s folds (Figure 2). The anterior chamber flare and cells increased. The corneal endothelial analysis revealed 922 cell/mm^2^. Therefore, the anterior chamber was irrigated with oxiglutatione solution to rinse out the venom on the third day after injury. The next day, the anterior chamber was clear. Fundus examination revealed a grade 2 vitreous opacity. Three days after irrigation (6 days after injury), stromal edema and Descemet’s membrane folds in the cornea increased and anterior chamber showed flare with uveitis again. B-scan ultrasonographic findings and fundus examination showed that vitreous opacity had increased. Therefore, we performed a 23-gauge sutureless vitrectomy to remove the vitreous opacity on day 6 after injury (Figure 3).

The anterior chamber inflammation and vitreous opacity were greatly resolved after surgery. Corneal endothelial cell density was 744 cell/mm^2^. The flashed electroretinography (ERG) result was normal in both eyes. The postoperative visual acuity of the operated eye was 7/10 with a normal fundus examination 2 months after injury. Aqueous and vitreous samples were not analyzed.

### Discussion

Stings from bees or wasps produce a variety of clinical manifestations [1-7]. In cases of corneal bee sting discussed in the literature, corneal complications have been reported most frequently [2-4]. In all these cases, the corneal edema regressed and infiltrates resulted in a corneal scar 4 to 6 weeks after removal of the stinger and the initiation of treatment. Corneal edema has been explained by cell death caused by activation of the complement cascade by proteins in the bee venom, leading to the production of anaphylotoxins and chemotactic factors [1,3]. Bee venom is a complex toxin having various impacts and components. It contains two types of biogenic amines: non-enzymatic polypeptide toxins (melittin, apamin, mast cell degranulating peptide) and enzymes (phospholipase A, phospholipase B, hyaluronidase). Ocular inflammation increases capillary protein permeability allowing greater leakage of proteins into the vitreous space.

The difference of wasp sting effects depends on the type of wasp venom involved and whether the venom was injected into the eye. The corneal edema found in cases of wasp sting generally ends with bullous keratopathy [7]. It has been noted that the cause of corneal edema in wasp stings is acetylcholine in the venom [1]. We observed decreased endothelial cell density in the left eye. This finding indicated that the wasp venom contained substances that were toxic to the corneal endothelial cells. In addition, Kitagawa et al. reported a wasp (hornet) sting-induced retinal damage with unrecordable ERG responses [6]. In our patient, ERG was normal after treatment. Unfortunately, we could not capture the species. A hornet sting of the cornea would have been more severe so it was excluded. This was verified following a medical interview of the patient’s companion. Together, the evidence indicated that the offending insect in this case was a paper wasp.

Wasp venom that had invaded through the wound caused inflammation of both the cornea and anterior chamber and also seemed to have passed through the open posterior capsule so that the vitreous was also *involved*. In our case, corneal edema and panuveitis deteriorated again in spite of anterior chamber irrigation. Toxins or chemotactic factors seemed to accumulate in the vitreous space.

It is difficult to differentiate sterile from infectious endophthalmitis, especially in cases of severe anterior inflammation induced by a toxic substance. Wasp venom may induce bacterial endophthalmitis concurrently with toxic injection. In our case, we did not analyze the anterior chamber aqueous or vitreous for diagnosis of bacterial endophthalmitis. If the clinical manifestation presented infectious endophthalmitis, empirical therapy, such as intravitreal antibiotics (i.e., vancomycin hydrochloride and ceftazidime) by intravitreal injection and/or systemic antibiotics are needed. Our case presented acutely without pain, whereas infectious endophthalmitis typically manifests acutely or subacutely with pain. The pathological condition resembled toxic anterior segment syndrome (TASS) or toxic endothelial cell destruction syndrome [8,9]. Therefore, we initially used topical dexamethasone sodium phosphate as a corticosteroid medication for post-traumatic inflammation therapy. Typically, TASS responds well to intense steroidal treatment. However, because steroid and anterior chamber irrigation did not respond well, it was necessary to clean up the toxic substance not only from the anterior chamber, but also from the vitreous space. Wasp venom was deemed to be the cause of panuveitis. Corneal endothelial cell density decreased rapidly despite the instillation of topical corticosteroid. Therefore, we decided to remove the causative substance rapidly from the vitreous space. Early vitrectomy is reported to be useful for the treatment of posterior uveitis [10] including debridement of the toxins they produce, removal of vitreous membranes, and clearing of vitreous opacity. In our case, early vitrectomy resulted in a good visual outcome.

### Conclusions

To our knowledge, improvement of wasp sting-induced panuveitis by vitrectomy has not been reported. Even though corneal wasp stings are not encountered frequently, the ophthalmologist should be aware of the potential complications that include not only corneal endothelial damage, but also panuveitis. Aggressive and early management may be required in the treatment of corneal wasp sting-induced complications.

## Competing interest

The authors declare that they have no competing interests.

## Authors’ contributions

YN drafted the manuscript. YN and AN carried out the operation and treatment. KS conceived of the study and participated in its design and coordination. All authors read and approved the final manuscript.
